# Patient’s awareness on COPD is the strongest predictor of persistence and adherence in treatment-naïve patients in real life: a prospective cohort study

**DOI:** 10.1186/s12890-021-01754-6

**Published:** 2021-11-27

**Authors:** Elsa López-Pintor, Justo Grau, Blanca Lumbreras

**Affiliations:** 1 Department of Engineering, Area of Pharmacy and Pharmaceutical Technologies, Miguel Hernández University, Crtra Alicante-Valencia km 81, Sant Joan d’Alacant, 03550 Alicante, Spain; 2 CIBER en Epidemiología y Salud Pública, Madrid, Spain; 3 Pneumology Department, General Hospital of Elche, Alicante, Spain; 4 Department of Public Health History of Science and Gynaecology, Miguel Hernández University, Crtra Alicante-Valencia km 81, Sant Joan d’Alacant, 03550 Alicante, Spain

**Keywords:** Chronic obstructive pulmonary disease, Adherence, Persistence, Inhaler medication, Treatment-naïve patients

## Abstract

**Background:**

There is little evidence about the factors that predict persistence/adherence in treatment-naïve patients with COPD in clinical practice. The aim of this study was to evaluate persistence and adherence levels among treatment-naïve patients diagnosed with COPD who had a prescribed inhaled medication, using data from real-world clinical practice.

**Methods:**

Multicentric study with a 6 month-followed-up period. Patients were considered persistent if they collected all their inhaler refills. In a random sample of patients, we evaluated adherence using the Test of Adherence to Inhalers (TAI). We assessed Health Related Quality of Life (HRQL) with St George's Respiratory Questionnaire (SGRQ).

**Results:**

Of the 114 patients included, 46 (40.4%) were defined as persistent. Patients who had awareness about COPD (adjusted RR 2.672, 95% CI 1.125–6.349) were more likely to be persistent; patients with multidose DPI were less likely to be persistent that those with single dose DPI (adjusted RR 0.341, 95% CI 0.133–0.877). Higher levels of SGRQ total were associated with a lower probability of persistence (adjusted RR 0.945, 95%CI 0.894–0.998). Patients who had had an appointment with their GP in the previous six months were more likely to be persistent (adjusted RR 3.107, 95% CI 1.022–9.466). Patients who had awareness about COPD and those with lower symptom SGQR score were more likely to be adherent (24/25, 96.0% vs 16/22, 72.7%, p = 0.025, and mean 29.1, sd 19.4 vs mean 41.4, sd 15.9, respectively, p = 0.026, respectively).

**Conclusions:**

Less than 50% of patients were defined as persistent. Patients’ awareness of their disease and levels of HRQL were associated with high rate of persistence and adherence. In addition, frequent visits to general practitioner, increases the rate of persistence to treatment.

**Supplementary Information:**

The online version contains supplementary material available at 10.1186/s12890-021-01754-6.

## Introduction

A central element in chronic obstructive pulmonary disease (COPD) management is the use of bronchodilators [1]. Each type presents different advantages and disadvantages regarding use and previous data showed that between 4 and 94% of patients exhibited incorrect inhaler usage [2]. Adherence to treatment (the extent to which a person take medication with the agreed recommendations from a clinician) and persistence (the act of continuing the treatment for the prescribed duration) are two relevant factors of the effectiveness of a treatment. Addressing both adherence and persistence brings deeper understanding of a patient’s medication-taking behavior [3], and this is particularly important in respiratory medicine, where many patients in do not use their inhalers correctly.

Lack of adherence and persistence in patients diagnosed with COPD have been widely described in both controlled clinical studies and those in clinical practice. However, because randomized controlled trials usually include homogeneous patient populations, they do not represent the real-world clinical population. Although adherence levels observed in clinical trials may be around 70–90%, levels of adherence in clinical practice are far lower (40–60%) [4, 5].

Many factors influence persistence and adherence to therapy, including patient’s age, Health Related Quality of Life (HRQL), comorbidities, concomitant medications, knowledge about the disease and treatment, complexity of the treatment, lack of training and education on inhaler use, among others [6–9]. HRQL is an essential factor included in the definition of patient-reported outcomes (PROs), measurements of any health status aspect directly indicated by the patient, which are often associated with overall treatment efficacy [10]. Most of the studies aiming to assess the relationship between HRQL and adherence have been carried out in patients who have had inhaler treatment during more than 6 months. In a recent study, the type of inhaler, patient awareness about the disease and treatment, and higher Health Related Quality of Life (HRQL) were associated with good adherence [4]. A previous systematic review showed however, that an improved HRQL may be a trigger for non-adherence in patients with COPD, and thus, the relationship between medication adherence and HRQL may be dual [11]. Nevertheless, there is little evidence about the influence of HRQL as a predictor factor of persistence and adherence in treatment-naïve patients. In addition, the majority of the studies are based on anonymized claims data in which patients cannot be evaluated regarding relevant factors such as knowledge and perception of their disease and treatment [12].

The aim of this study was to evaluate persistence and adherence levels among treatment-naïve patients diagnosed with COPD who had a prescribed inhaled medication, using data from clinical practice.

## Methods

### Study design

Multi center prospective cohort study of treatment-naïve patients using COPD medications.

### Population

A sample of consecutive treatment-naïve patients diagnosed with COPD were invited to participate in the study when they attended the pneumology department in the General Hospital of Elche, Hospital Sant Joan d’Alacant and General Hospital of Alicante, Spain, from March 2018 to January 2019. All the patients who were invited to participate in the study agreed to be included. Diagnosis was established after a spirometry test was carried out (ICD J40-J47). Only patients with no prior use of inhaler device were included. The exclusion criteria have previously been defined [4].

The majority of the population in Spain uses the National Health Care System as the main medical service in which prescription costs are mainly covered. In order to have access to patients’ medical records we only included patients who used this system and therefore, the insurance status is not a relevant variable in this study.

We defined the index date as the date of a patient’s first prescription claim for a COPD drug of interest and patients were followed-up for 6 months through the pharmaceutical electronic register. Given the relatively short follow-up period, the 114 patients used the same inhaler during the six subsequent refills.

### Data collection

#### Outcome variables

Persistence was assessed through the evaluation of the six subsequent refills of the inclusion inhaler, (pharmaceutical electronic register). In order to calculate the drug use as a continuous variable, we assessed the total number of doses dispensed from therapy initiation divided by the prescribed daily dose. A patient was considered “persistent” if he/she had refilled the six prescriptions of the inhaler, and “non-persistent” if at least one of the prescriptions was missed. This binary variable (persistence yes/no) was the main outcome variable to assess persistence in our study.

In addition, we evaluated the different persistence profile for each patient to assess the different patient’s behavior. Hence, we evaluated patients according to the three components previously described [13]: initiation (the patient takes the first dose of a prescribed medication), implementation (from initiation until the last dose is taken) and discontinuation (the end of the therapy). We classified the patients into: late or non-initiation of the prescribed treatment (those patients who did not take the first dose of the prescribed medication and they initiated the treatment after the second prescription or later), sub-optimal implementation of the dosing regimen (those patients who initiated the treatment but they did not regularly collect their prescription) and early discontinuation of the treatment (those patients who omitted the next dose to be taken).

We also calculated persistence as a continuous variable (days of persistence), considering the number of days in the follow-up period, according to a previous study [14].

We assessed adherence in a random sample of patients at the end of the period of study. Adherence was assessed by phone through a self-reported method, the Test of Adherence to Inhalers’ (TAI) [15], which includes 12 questions classified into two main domains, the patient (items #1 to #10) and the health professional (items #11 and #12) domain. We used the 10-items TAI whose total score ranges from 10–50. Adherence is rated as good (score: 50), intermediate (score: 46–49), or poor (score: < 45). We classified patients in two groups, above and below the mean value of TAI score in the studied population. We also estimated adherence as a continuous variable (TAI score).

As we previously did [4], adherence and persistence were based on the primary inhaler, which was determined by the treating physician.

#### Independent variables

The following variables were assessed at the entry of the study before the initiation of the treatment.

We collected, through a standard questionnaire (Additional file 1): 1) sociodemographic variables: age, sex, educational level, and patients’ employment status (employed worker, self-employed worker, retired, unemployed; 2) clinical variables: smoking habit and physical activity. Physical activity was assessed through the following questions: Do you think you are active or sedentary?; Do you practice physical activity? (sometimes, almost always, always or never); Physical activity frequency (every day, once or twice/week, between 3 and 5 times/week or never). According to patients’ answers, we classified the patients into active or sedentary and the frequency of physical activity.

We also assessed whether patients received explanation about treatment and patients’ variables related with their awareness and perceptions on disease and its seriousness. In order to classify if a patient had awareness about COPD we included the following questions in the questionnaire: Do you know if you have COPD? (Yes, I suffer from this disease, No, I have another disease, Don’t know/no opinion); Do you know if COPD is a chronic disease? (Yes, No, Don’t know/no opinion); Do you know if COPD is a serious disease? (No, COPD is a mild disease, Yes, COPD is a serious/very serious disease, Don’t know/no opinion). A patient was classified as having awareness if he/she answered yes to all three questions.

b) We also collected other clinical variables through electronical medical records in the index data: patient’s COPD characteristics according to the Global Initiative for Obstructive Lung Disease (GOLD) [1]; number of visits to Emergency Services for breathing related problems in the last 12 months and frequency of medical appointments with the general practitioner. We also retrieved Body Mass Index (BMI) and treatment-related variables: type of inhaler device (single dose Dry Powder Inhalers (DPI), multidose DPI, Soft Mist Inhaler (SMI) and Metered dose inhalers (MDI)) and number of inhalers.

We addressed the following agents: inhaled corticosteroids (ICS), combination long-acting (LABA) beta2-agonists/ICS in one device, long-acting (LAMA) anticholinergics, combination LAMA/LABA in one device, and triple combination LAMA/LABA/ICS in one device. The classification of single-dose or multi-dose DPI depend on how the dry powder is stored, and it does not refer to the number of doses per inhalation o the combination of different agents [16].

Patients also completed the St George's Respiratory Questionnaire (SGRQ) [17]. The SGRQ is the most widely used qualified COPD-specific instrument that has 50 items comprising three domains: symptoms (eight items), activity (16 items), and impact (psychosocial) (26 items). Scores range from 0 (no impairment) to 100 (maximum impairment). A mean change score of 4 units is associated with slightly efficacious treatment, 8 units for moderately efficacious change and 12 units for very efficacious treatment [18]. According to a previous study in Spanish general population, overall mean SGRQ scale score was 8.4 (sd 11.3) [19]. In a previous study [4], the different mean SGRQ scale scores for patients with severe/very severe COPD vs patients with mild/moderate COPD were: symptoms: 44.2 (sd 22.2) vs 36.9 (sd 20.4); activity: 62.6 (sd 26.6) vs 48.7 (sd 22.3); impact: 42.8 (sd 16.4) vs 36.4 (sd 14.7), and total: 49.4 (sd 18.0) vs 40.4 (sd 14.9).

### Sample size

According to previous studies [12], 10% of patients were defined as non-persistent. They defined non persistence as a treatment gap of > 90 days and > 180 days in sensitivity analysis (binary persistence yes/no). Given the shorter period of follow-up in our study (6 months), we defined non persistence as a treatment gap of > 30 days (if the patient failed to collect any of the 6 prescriptions). Therefore, we will need to include a total of 112 patients (5% precision and 95% confidence interval).

### Statistical analysis

All data were computerised anonymously and checked to discard errors. Statistical precision was determined through the calculation of 95% confidence intervals using the appropriate method according to the type of measurement and the available data. All analyses carried out with the statistical programme Stata/SE (Stata Corp., College Station, Texas, USA).

We estimated the incidence of persistence according to relevant variables. The effect of potential variables predicting 6-month non persistence to therapy on a patient level was considered by means of a stratified analysis a Poisson regression (95% confidence intervals). For the multivariable regression models, a backward elimination methodology was used (the model included only predictors that reached statistical significance p < 0.005).

## Results

### Baseline patients’ characteristics

A total of 114 patients met the criteria for inclusion in the study (Table 1). Of the 114 patients, 63 (55.3) belong to the General Hospital of Elche, 30 (26.3%) to the Hospital Sant Joan d’Alacant, and 21 (18.4%) to the General Hospital of Alicante. The mean age was 65.9 years old (standard deviation (sd) 10.7), 77 (67.5%) were men and 22 (19.3%) patients did not have studies. More than half of patients were former or never smokers (63, 55.3%) and 94 (82.5%) had mild or moderate COPD (Table 2). Mean total SGQR score was 39.2 (sd 14.9); mean symptoms SGQR score was 36.5 (sd 19.5); mean activity SGQR score was 45.5 (sd 23.3), and mean impact SGQR score was 36.2 (sd 13.9). The majority of patients (38, 57.6%) usually had an appointment with their General Practitioner (GP) each 6 months or less. Of the 114 patients included, 58 (50.9%) had awareness about COPD seriousness; 53 (46.5%) had awareness about COPD chronicity. In addition, 55 (48.2%) had received explanation about their treatment.

**Table 1:** Description of the study patients and the relationship between the sociodemographic and activity variables with persistence to inhalers

Variables	Persistence		
	Yes (46, 40.4%)	No (68, 59.6%)	p value
Sex			0.977
Men	31 (67.4)	46 (67.6)	
Women	15 (32.6)	22 (32.4)	
Age (mean, sd)	66.28 (10.6)	65.69 (10.8)	0.774
Education level			0.843
Without studies	10 (23.3)	12 (18.2)	
Primary studies	19 (44.2)	28 (42.4)	
Secondary studies	10 (23.3)	17 (25.8)	
University studies	4 (9.3)	9 (13.6)	
Employment situation			0.573
Employed worker	13 (28.3)	12 (17.6)	
Own-account worker	4 (8.7)	8 (11.8)	
Retired	25 (54.3)	40 (58.8)	
Unemployed	4 (8.7)	8 (11.8)	
BMI (median, range)	26.9 (4.8)	28.2 (5.3)	0.213
BMI			0.238
< 25	14 (30.4)	23 (33.8)	
25–30	23 (50.0)	24 (35.3)	
> 30	9 (19.6)	21 (30.9)	
Physical activity			0.644
Active	32 (69.6)	50 (73.5)	
Sedentary	14 (30.4)	18 (26.5)	
Frequency of physical activity (in active patients)			0.699
Every day	16 (34.8)	28 (41.2)	
1–2/week	14 (30.4)	21 (30.9)	
3–5/month	7 (15.2)	11 (16.2)	
Never	9 (19.6)	8 (11.8)	
Smoking behaviour			0.032
Current	15 (32.6)	36 (52.9)	
Former and never smoker	31 (67.4)	32 (47.1)	
Smoking pack/year (median, range)	32.38 (27.99)	33.78 (21.53)	0.773

*BMI* body mass index, *sd* standard deviation

**Table 2:** Description of the study patients and the relationship between the clinical variables with persistence to inhalers

Variables	Persistence		
	Yes (46, 40.4%)	No (68, 59.6%)	p value
Visits to GP			0.040
≤ 6 months	19 (73.1)	19 (47.5)	
> 6 months	7 (26.9)	21 (52.5)	
Visits to the Emergency Service in the last 12 months			0.361
No	36 (78.3)	48 (70.6)	
Yes	10 (21.7)	20 (29.4)	
COPD			0.535
Mild + moderate	35 (85.4)	59 (89.4)	
Severe + very severe	6 (14.6)	7 (10.6)	
Knowledge about COPD severity and chronicity			0.011
No	18 (39.1)	43 (63.2)	
Yes	28 (60.9)	25 (36.8)	
To receive explanation about treatment			0.223
No	27 (58.7)	32 (47.1)	
Yes	19 (41.3)	36 (52.9)	
Number of inhalers			0.110
1	32 (69.6)	56 (82.4)	
2/3	14 (30.4)	12 (17.6)	
Health related quality of life (mean, sd)			
Symptoms	35.7 (27.2)	46.7 (20.4)	0.522
Activity	38.9 (27.2)	46.7 (20.4)	0.026
Impact	43.9 (27.2)	46.7 (20.4)	0.227
Total	36.0 (17.1)	41.4 (13.4)	0.023
Inhaler device types			0.021
Single dose DPI	21 (42.9)	28 (57.1)	
Multidose DPI	10 (25.6)	29 (74.4)	
SMI	8 (57.1)	6 (42.9)	
MDI	7 (58.3)	5 (41.7)	

*DP*I dry powder inhalers, *SMI* soft mist inhaler, *MDI* metered dose inhalers, *GP* general practitioner, *COPD* chronic obstructive pulmonary disease

### Patients’ persistence profile

**Fig. 1:**
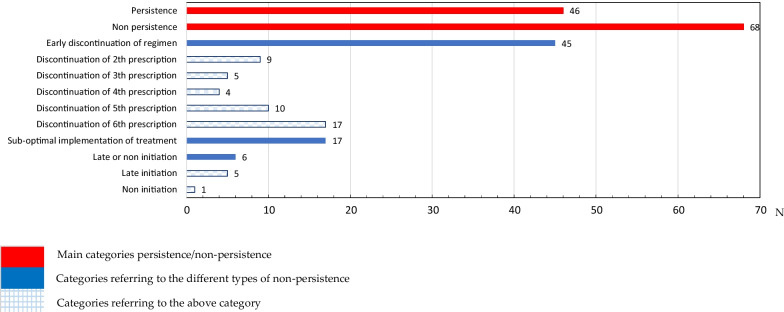
Description of the number of patients included in the study according to their profile when collecting their prescription

According to Fig. 1, patients’ persistence profiles were divided into different categories. Out of the 114 patients included in the study, 46 (40.4%) were defined as persistent. Of the 68 (59.6%) patients classified as non-persistent, 45/68 (66.2%) discontinued their treatment early, 17/68 (25.0%) had a sub-optimal implementation of the dosing regimen, and d) 6/68 (8.8%) started late or did not initiate their prescribed treatment (1 patient did not initiate the treatment). Of the 45 patients who discontinued their regimen early, most discontinued it before the 6th prescription (17/45, 37.8%) or before the 5th prescription (10/45, 22.2%).

### Description of the variables associated with persistence

Those former and never smoker patients (31, 67.4%) were more likely to be persistent than those current smokers (15, 32.6%), p = 0.032 (Table 1). Those patients who had had an appointment with their GP in the previous six months were more likely to be persistent (19, 73.7% vs 7, 26.9%, p = 0.040). Those patients who had awareness about COPD seriousness and chronicity were more likely to be persistent (28, 60.8% vs 18, 39.1%), p = 0.011 (Table 2).

**Fig. 2:**
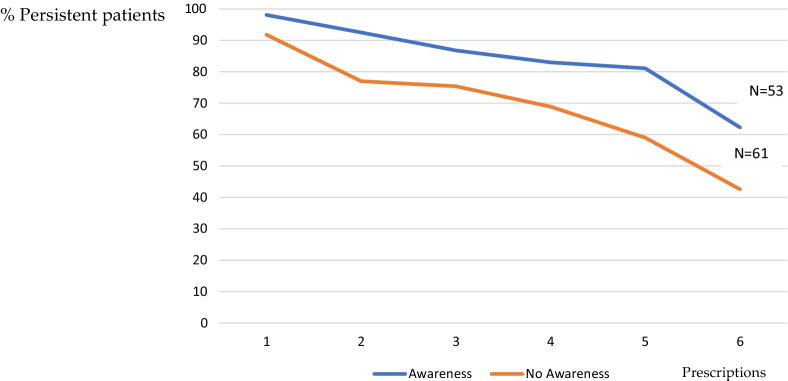
Percentage of persistent patients according to patients’ awareness about COPD severity and chronicity along the period of study

Figure 2 shows the different persistence rates between those patients who had awareness about COPD seriousness and chronicity and those who not along the period of study.

Of the 61 patients who did not have awareness about COPD seriousness and chronicity, 18 (39.1%) were classified as non-persistent, 26 (57.8%) early discontinued their treatment, 12 (70.6%) had a sub-optimal implementation of the dosing regimen, and 5 (83.3%) started late or did not initiate their prescribed treatment (p = 0.040).

Total SGRQ score was significantly higher in non-persistent patients than in those persistent patients (mean 41.4, sd 13.4 and mean 36.0, sd 17.1, respectively, p = 0.023). There were also differences according to activity SGQR score in non-persistent patients vs persistent patients (mean 46.7, sd 20.4 and mean 38.9, sd 27.2, respectively, p = 0.026) (Table 2).

According to inhaler device (Table 2), those patients using a single dose DPI (42.9%), SMI (57.1%) and MDI (58.3%) were more likely to be persistent than those using a multidose DPI (25.6%), p = 0.021. According to persistence categories, patients with multidose DPI were more likely to start late or did not initiate their prescribed treatment than patients with other inhaler device types (3, 7.7% vs 3, 4.0%) and to have a sub-optimal implementation of treatment (20, 51.3% vs 25, 33.3%), p = 0.021.

In multivariate analysis (adjusted by SGRQ total and activity, awareness about COPD seriousness and chronicity, visits to GP in the previous 6 months, smoking behaviour and type of inhaler), those patients who had awareness about COPD (adjusted RR 2.672, 95% CI 1.125–6.349, p = 0.026) and those patients who had had an appointment with their GP in the previous six months (adjusted RR 3.107, 95% CI 1.022–9.466, p = 0.046) were more likely to be persistent. Higher levels of SGRQ total were associated with a lower probability of persistence (adjusted RR 0.945, 95%CI 0.894–0.998, p = 0.041). Those patients with Multidose DPI were more likely to be no persistent that those with other inhaler devices (adjusted RR 0.341, 95% CI 0.133–0.877, p = 0.040) (Table 3).

**Table 3:** Multivariate analysis assessing the relationship between different variables and probability of persistence

Variables	RR	CI 95%	p value
Health related quality of life			
Activity	0.972	0.934–1.011	0.161
Total	0.945	0.894–0.998	0.041
Awareness about COPD severity and chronicity			
No	1.00		
Yes	2.672	1.125–6.349	0.026
Visits to GP			
> 6 months	1.00		
≤ 6 months	3.107	1.022–9.446	0.046
Smoking behaviour			
Current	1.00		
Former and never smoker	2.515	1.010–6.260	0.048
Inhaler device type			
Other device	1.00		
Multi DPI	0.341	0.133–0.877	0.026

Mean days of persistence was 104.9 (sd 56.3). Mean days of persistence was lower in patients who did not have awareness about COPD (94.1, sd 58.7) in comparison with patients who related to have awareness about the disease (mean 116.6, sd 51.6), p = 0.035. Mean days of persistence were lower in patients using multidose DPI (89.3, sd 46.6) in comparison with patients using other inhaler devices, p = 0.036 (Additional file 1).

### Adherence to treatment

Out of 114 patients, 47 (41.2%) were randomly selected to be evaluated about their adherence to inhaler device. There were no differences between those who answered the TAI test and those who not. Of the 47 patients, 27 (57.4%) were classified as good adherent, 13 (27.7%) as intermediate adherent and 7 (14.9%) as poor adherent. Mean (sd) followed-up period until the TAI questionnaire was carried out was 122 days (36.4) and there were not differences according to the different categories (good/intermediate/poor) (p = 0.308) (data not shown).

Mean TAI punctuation was 44.4 (sd. 8.2). Those patients with good/intermediate adherence were more likely to be classified as persistent (16/40, 40.0%) or to have an early discontinuation of regimen (18/40, 45.0%) than those with poor adherence (1/7, 14.3% and 2/7, 28.6%, respectively) (p = 0.031). Thus, patients who regularly took their medication (even if they stopped it earlier) were more likely to consider themselves as adherent patients than if they had a sub-optimal implementation of treatment or started it later.

Those patients who had awareness about COPD seriousness and chronicity were more likely to be adherent (24/25, 96.0%% vs 16/22, 72.7%), p = 0.025. Symptom SGQR score was significantly lower in patients with good adherence than in those with poor/intermediate adherence (mean 29.08, sd 19.43 and mean 41.35, sd 15.85, respectively, p = 0.026).

## Discussion

In our study, only 40% of the treatment-naïve COPD patients had continued their treatment after 6 months of follow-up and 57% of the patients interviewed were classified as good adherent. In contrast with previous studies [8], we showed a correlation between persistence and adherence. Patients who did not have awareness about COPD, lower HRQL and patients with multidose DPI were more likely to be no persistent. In addition, patients who had awareness about COPD and those with lower symptom SGQR score were more likely to be adherent.

Our results showed a lower rate of persistence than previous studies. In a study in the United Kingdom, three quarters of newly diagnosed COPD patients continued their treatment after 6 months [20]. Nevertheless, the authors acknowledged the lack of an accurate diagnosis of COPD and a probable misclassification of drug exposure. In contrast, previous research found that only 12–27% of naïve-treatment patients taking drugs other than tiotropium persisted in treatment after 6 months [21].

Out of the 68 patients who were non-persistent, 45 (66.2%) had an early discontinuation of regimen. Hence, this pattern of early discontinuation indicates a lack of willingness to take the medication for more than a few months (40% of the patients discontinued the treatment before the fourth month). In addition, there was a relevant percentage of patients who showed a sub-optimal implementation of treatment and 5% of all patients either had a late initiation or did not initiate treatment. This has had an impact on COPD control and leads to the wasting of health-care resources burden [22]. Efforts should be made to improve this pattern when a patient initiates inhaler therapy, and health professionals should be aware of the factors that negatively influence persistence.

Fewer than 50% of patients admitted to having awareness about COPD seriousness and chronicity. This awareness regarding the disease was found to be the most powerful predictor of persistence and adherence to inhalation therapy, which has also been referred to by other authors [23, 24]. Subjects that were knowledgeable about COPD characteristics and those who had recently had an appointment with their GP showed a higher rate of persistence to treatment. Patients’ HRQL in baseline was also associated with persistence. COPD education strategies involving primary care centres need to be modified to achieve improved patient outcomes.

Persistent patients and those with good adherence showed in the baseline, higher levels of HRQL. Although evidence on the relationship between medication persistence/adherence in treatment-naïve patients is lacking, there are controversial data about the relationship between HRQL and adherence/persistence in experienced patients. Adherence can affect HRQL, but HRQL may also impact on medication adherence. A previous systematic review including studies in experienced patients showed a dual association between medication adherence and HRQL: the effect of medication adherence on HRQL might be a consequence of the effectiveness of the therapy, but an increased HRQL could be a trigger for non-adherence in subjects with COPD [11]. However, in another study which included patients with a chronic disease such as hypertension, when HRQL increased, the level of adherence to therapeutic recommendations also did [25]. HRQL can be affected by the existence of a chronic disease, its chronic nature and factors related with the negative impact of the disease on the patient’s physical, emotional and social wellbeing [26]. Therefore, HRQL can affect the person’s attitude towards the disease, as well as the attitude to treatment, which includes the appropriate level of adherence [27]. The inclusion of HRQL in clinical studies, as a relevant patient-reported outcome (PRO), provide important supporting evidence of factors related to persistence and adherence to treatment. The European Medicines Agency (EMA) and the US FDA consider patients’ perspective as an important aspect of drug approval [28, 29].

Device characteristics were also associated with different persistence rates. There is a growing availability of different types of medication and inhaler devices which constitute additional key factors in patients’ compliance with their medical therapy. More evidence is required to assist clinicians in prescribing the optimal medication, given that as many as 94% of patients made at least one error when they used the inhaler device [30]. In our study, although medication persistence was low for all the types of inhalers, patients in treatment with multidose DPI were less likely to be persistent. To our knowledge, this is the first study describing the relationship between inhaler devices and persistence in treatment-naïve patients with COPD which includes an analysis of patients’ persistence pattern. A previous retrospective cohort study did not show impact of inhaler device (multiple-dose versus single-dose inhaler) on COPD patients’ persistence [31]. However, the above study included some limitations related with the existence of an intensive monitoring and lung rehabilitation program in the study setting, and the lack of an ac-curacy diagnosis on the database (some asthma patients could have been classified as COPD patients).

This study has some limitations. First, and due to the longitudinal design, the observational period was limited to 6 months. Secondly, the sample size was small because of the need to evaluate patients’ awareness and perceptions about COPD and treatment. There is little evidence on persistence and adherence regarding treatment-naïve patients. We based the sample size estimation on a previous retrospective study with a 12-month followed-up period [11]. They defined non persistence as a treatment gap of > 90 days and > 180 days in sensitivity analysis (binary persistence yes/no). Given the shorter period of follow-up in our study (6 months), we defined non persistence as a treatment gap of > 30 days (if the patient failed to collect any of the 6 prescriptions). However, we included all the consecutive treatment-naïve patients who were diagnosed with COPD, excluding a selection bias, and increasing the external validity of the study (together with the inclusion of patients from different centres).

In addition, although we could only interview a random sample of patients about their adherence to treatment, patients who were interviewed had similar sociodemographic and clinical characteristics to those who were not.

We classified patients’ awareness about COPD considering three different questions to cover the description of patients’ awareness. The questionnaire applied to our study was previously used in a similar population in which the questionnaire was previously piloted to ensure patients understood the questions and the responses were not ambiguous [4]. As we previously did in that study, we decided to combinate the three questions related with awareness about COPD severity and chronicity, because there was a high concordance between them. In the present study, those patients who answered they knew they have COPD were more likely to know that COPD was a chronic disease (72.7%) and to know that COPD was a serious disease (77.3%). Therefore, we considered that this question included several aspects related with patients’ knowledge on COPD characteristics. In the literature, there are several questionnaires to assess patient’s awareness on COPD with different levels of accuracy [32], which have been carried out in different settings [33]. We decided to use the three questions because similar aspects had been previously asked to assess patients’ awareness on COPD [34]. We used these three questions with three options, ‘yes/no/don’t know’, which make them less time-consuming for patients, easier to score, and therefore suitable for use in this research. In addition, the results of this classification can be considered reliable since we showed not only differences between patients’ awareness about COPD seriousness and chronicity and persistence, but also a relationship between mean months of persistence and patients’ awareness.

Although previous evidence showed that GP’s emphasis on the importance of the medication could be a relevant facilitator of patients’ persistence [35], we did not consider it. Patients included in this study were recruited in the Pneumology Services when they were firstly prescribed an inhaler. Thus, the person who explained the diagnosis and the new treatment was the pneumologist, who is specialized in the respiratory system. Previous evidence stated that patients controlled by a pneumologist were less likely to perform the inhalation manoeuvers incorrectly in comparison with those controlled by a GP [36]. Thus, this factor would not have had a great impact on our results. We classified the patients according to the Global Initiative for Obstructive Lung Disease. However, we did not have access to spirometry data referring seven of the patients to classify the patients’ disease. We did not use the ABCD assessment tool that was updated in 2017. We started the study in 2018 but the protocol was approved by the different committees and authorities before the update of the guidelines. In multivariable analysis, we found no association between smoking behaviour and adherence to treatment. Previous evidence showed that smoking and highly educated young patients were associated with poor adherence to treatment when inhaler overuse was defined as poor adherence [37]. However, we did not include overuse as an aspect of adherence to treatment and the TAI questionnaire only detects underuse of inhalers.

The strength of this study lies in the fact that it is a prospective design in real-world practice with unselected population. Previous studies [38] have included extensive databases to evaluate aspects such as daily dosing frequency and adherence in treatment-naïve patients. However, this is the first study in which naïve-treatment patients’ awareness on both disease and treatment and impact of HRQL on persistence/adherence were evaluated.

## Conclusions

In conclusion, given the low rate of persistence and adherence in treatment-naïve patients with COPD, it is essential to know which variables can predict this lack of compliance with their treatment. Raising patient awareness of the disease and improving cooperation between patients and clinicians should be implemented in practice before the choice of the inhaler to increase effectiveness of the treatment.

## Supplementary Information

**Additional file 1. Fig. S1**: Questionnaire to collect patients’ sociodemographic and clinical variables at the entry of the study before the initiation of the treatment. **Table S1**: Description of the study patients and the relationship between the sociodemographic and activity variables with adherence and persistence to inhalers (as a continuous variables). **Table S2**: Description of the study patients and the relationship between the clinical variables with adherence and persistence to inhalers (as a continuous variables).

## Data Availability

The datasets used and/or analysed during the current study are available from the corresponding author on reasonable request.

## Abbreviations

COPD: Chronic obstructive pulmonary disease
GP: General practitioner
HRQL: Health Related Quality of Life
sd: Standard deviation
SGRQ: St George's Respiratory Questionnaire
TAI: Test of Adherence to Inhalers
GOL: Global Initiative for Obstructive Lung Disease
BMI: Body mass index
DPI: Dry powder inhalers
SMI: Soft mist inhaler
MDI: Metered dose inhalers
ICS: Inhaled corticosteroids
LABA: Combination long-acting
LAMA: Long-acting anticholinergics
FDA: Food and Drug Administration
EMEA: European Medicines Agency
CI: Confidence interval
