# Correction to: Patient’s awareness on COPD is the strongest predictor of persistence and adherence in treatment-naïve patients in real life: a prospective cohort study

**DOI:** 10.1186/s12890-022-01857-8

**Published:** 2022-03-07

**Authors:** Elsa López-Pintor, Justo Grau, Blanca Lumbreras

**Affiliations:** 1 Department of Engineering, Area of Pharmacy and Pharmaceutical Technologies, Miguel Hernández University, Crtra Alicante-Valencia km 81, Sant Joan d’Alacant, 03550 Alicante, Spain; 2 CIBER en Epidemiología y Salud Pública, Madrid, Spain; 3 Pneumology Department, General Hospital of Elche, Alicante, Spain; 4 Department of Public Health History of Science and Gynaecology, Miguel Hernández University, Crtra Alicante-Valencia km 81, Sant Joan d’Alacant, 03550 Alicante, Spain

## Correction to: BMC Pulmonary Medicine (2021) 21:388 10.1186/s12890-021-01754-6

Following publication of the article [1], the authors flagged that the first and last names of the authors had been erroneously swapped in the author list.

The author list has since been corrected in the published article and the corrected author list may be seen in this correction.
