# Supplementary material for: Patient’s awareness on COPD is the strongest predictor of persistence and adherence in treatment-naïve patients in real life: a prospective cohort study
Source: BMC Pulm Med. 2021 Nov 27;21:388. doi: 10.1186/s12890-021-01754-6 (PMC8627039; doi:10.1186/s12890-021-01754-6)
Supplement: Supplementary file 1 — Additional file 1. Fig. S1: Questionnaire to collect patients’ sociodemographic and clinical variables at the entry of the study before the initiation of the treatment. Table S1: Description of the study patients and the relationship between the sociodemographic and activity variables with adherence and persistence to inhalers (as a continuous variables). Table S2: Description of the study patients and the relationship between the clinical variables with adherence and persistence to inhalers (as a continuous variables). [file 12890_2021_1754_MOESM1_ESM.docx]

**Figure 1. Questionnaire to collect patients’ sociodemographic and clinical variables.**

**Sex**: 1) Man 2) Woman

**Date of Birth**: (dd /mm /aa): ……………………….

**Educational level**

1) Without studies 2) Primary studies 3) Secondary studies 4) University studies

**Employment situation:**

1) Employed worker 2) Own-account worker 3) Retired 4) Unemployed

**You consider yourself…**

1) Active 2) Sedentary

**In relation to your previous answer, how often do you do it?**

1)Every day 2) once or twice a week 3) between 3-5 times a week 4) Never

| **SMOKING HABIT** |  | **QUESTION** |  |
| --- | --- | --- | --- |
| **Are you a smoker?**  (At present) |  | How many cigarettes do you smoke per day? |  |
|  |  | How many years have you been smoking? |  |
| **Are you an ex-smoker?** |  | How many cigarettes did you smoke per day? |  |
|  |  | How many years did you smoke in total? |  |
|  |  | How long ago did you give up smoking? |  |
| At what age did you start to smoke? (smokers and ex-smokers) | | ………… aproximate age |  |
| **I have never smoked** |  |  | |

**QUESTIONS ABOUT SMOKING HABIT**

**QUESTIONS ABOUT PATIENTS’ KNOWLEDGE AND INFORMATION**

| 1. Do you know if you have a Chronic Obstructive Pulmonary Disease, COPD? | 1. Yes, I have this condition  2. No, I have another, called ………………………  3. Don´t know/no answer |
| --- | --- |
| 1. Do you know if it is a chronic condition (lifelong disease) | 1. YES 2. NO 3. Don´t know/no answer |
| 1. How would you describe it in terms of severity? | 1. It is a mild disease  2. It is a serious illness  3. It is a very serious disease  4. Don´t know/no answer |
| 1. Have you been told how to use the inhaler? | 1. Yes  2. No |

**Table 1. Description of the study patients and the relationship between the sociodemographic and activity variables with adherence and persistence to inhalers (as a continuous variables).**

|  | **Adherence** | | **Persistence (days)** | |
| --- | --- | --- | --- | --- |
|  | **Mean (sd)** | **p-value** | **Mean (sd)** | **p-value** |
| **Sex** |  | 0.145 |  | 0.669 |
| Men | 45.42 (27.9) |  | 103.4 (52.7) |  |
| Women | 41.42 (27.0) |  | 108.4 (64.4) |  |
| **Education level** |  | 0.503 |  | 0.067 |
| Without studies | 44.8 (4.9) |  | 112.8 (50.2) |  |
| Primary studies | 42.3 (10.9) |  | 112.8 (58.8) |  |
| Secondary studies | 45.9 (4.7) |  | 98.2 (50.6) |  |
| University studies | 47.2 (4.6) |  | 67.7 (65.2) |  |
| **Employment situation** |  | 0.332 |  | 0.556 |
| Employed worker | 41.2 (12.9) |  | 100.9 (53.4) |  |
| Own-account worker | 47.8 (3.5) |  | 99.4 (47.4) |  |
| Retired | 45.6 (4.8) |  | 111.3 (58.8) |  |
| Unemployed | 44.3 (6.9) |  | 87.2 (61.2) |  |
| **BMI** |  | 0.303 |  | 0.796 |
| - <25 | 47.2 (4.0) |  | 99.8 (59.2) |  |
| - 25-30 | 42.6 (11.1) |  | 107.9 (52.5) |  |
| - >30 | 44.5 (5.8) |  | 106.8 (59.7) |  |
| **Physical activity** |  | 0.399 |  |  |
| - Active | 43.8 (9.2) |  | 107.1 (56.7) |  |
| - Sedentary | 46.2 (4.0) |  | 98.5 (55.3) |  |
| **Frequency of physical activity (in active patients)** |  | 0.347 |  | 0.502 |
| Every day | 45.3 (5.5) |  | 103.7 (50.4) |  |
| 1-2/week | 44.9 (5.7) |  | 103.0 (48.9) |  |
| 3-5/month | 40.0 (15.7) |  | 122.2 (80.1) |  |
| Never | 46.2 (3.9) |  | 98.5 (55.3) |  |
| **Smoking behaviour** |  | 0.072 |  | 0.078 |
| Current | 41.7 (26.4) |  | 94.8 (48.3) |  |
| Former and never smoker | 46.1 (4.7) |  | 113.7 (61.5) |  |

BMI: Body Mass Index; sd: standard deviation.

**Table 2. Description of the study patients and the relationship between the clinical variables with adherence and persistence to inhalers (as a continuous variables).**

|  | **Adherence** | | **Persistence (days)** | |
| --- | --- | --- | --- | --- |
|  | **Mean (sd)** | **p-value** | **Mean (sd)** | **p-value** |
| **Visits to GP** |  | 0.722 |  | 0.854 |
| =<6 months | 44.6 (27.6) |  | 106.3 (55.9) |  |
| >6 months | 43.8 (5.4) |  | 104.2 (56.9) |  |
| **Visits to the Emergency Service in the last 12 months** |  | 0.879 |  | 0.733 |
| No | 44.6 (4.8) |  | 103.3 (54.7) |  |
| Yes | 44.1 (9.1) |  | 107.6 (63.4) |  |
| **COPD** |  | 0.724 |  | 0.709 |
| Mild + moderate | 44.1 (8.8) |  | 102.6 (55.9) |  |
| Severe+ very severe | 45.3 (5.1) |  | 108.6 (41.9) |  |
| **Awareness about COPD severity and chronicity** |  | 0.032 |  | 0.035 |
| No | 40.1 (6.1) |  | 94.1 (58.7) |  |
| Yes | 49.0 (4.9) |  | 116.6 (51.6) |  |
| **To receive explanation about treatment** |  | 0.044 |  | 0.368 |
| No | 36.1 (12.0) |  | 96.6 (49.9) |  |
| Yes | 45.7 (29.0) |  | 107.5 (68.3) |  |
| **Number of inhalers** |  | 0.973 |  | 0.636 |
| 1 | 44.4 (27.6) |  | 103.5 (54.9) |  |
| 2/3 | 44.3 (5.0) |  | 109.6 (61.8) |  |
| **Type of inhaler** |  | 0.560 |  | 0.036 |
| Single dose DPI | 40.9 (17.1) |  | 104.1 (52.5) |  |
| Multidose DPI | 46.5 (4.9) |  | 89.3 (46.6) |  |
| SMI | 45.7 (4.9) |  | 137.5 (84.3) |  |
| MDI | 46.0 (4.3) |  | 104.9 (56.3) |  |

DPI: Dry Powder Inhalers, SMI: Soft Mist Inhaler, MDI: Metered dose inhalers.

GP: General Practitioner; COPD: Chronic Obstructive Pulmonary Disease.
